# Complete Genome Sequence of Vibrio alginolyticus ATCC 17749^T^

**DOI:** 10.1128/genomeA.01500-14

**Published:** 2015-01-29

**Authors:** Xiao-Fei Liu, Yuan Cao, He-Lin Zhang, Ying-Jian Chen, Cheng-Jin Hu

**Affiliations:** Department of Laboratory Medicine, Jinan Military General Hospital, Jinan, Shandong, China

## Abstract

Vibrio alginolyticus is a Gram-negative halophilic bacterium and has been recognized as an opportunistic pathogen in both humans and marine animals. It is the causative agent of food-borne diseases, such as gastroenteritis, and it invades through wounds in predisposed individuals. In this study, we present the completed genome of V. alginolyticus ATCC 17749^T^ through high-throughput sequencing.

## GENOME ANNOUNCEMENT

Vibrio species are widely distributed in aquatic environments (1). Vibrio alginolyticus is a Gram-negative halophilic bacterium with worldwide distribution (2). On the coast of southern China, V. alginolyticus is the dominant Vibrio species found both in seawater and in farmed marine animals (1, 3). Although V. alginolyticus is most commonly associated with wound infections, otitis media, and otitis externa (4, 5), it is increasingly recognized as an important intestinal pathogen in humans. In 1980, Hiratsuka, Saitoh, and Yamane (6) isolated the first V. alginolyticus from a patient with acute enterocolitis. It mainly affects persons who have had direct contact with seawater or those who have handled shellfish (7).

V. alginolyticus strain ATCC 17749^T^ was isolated from spoiled horse mackerel, which caused food poisoning; the strain was kindly provided by the Marine Culture Collection of China (Xiamen, Fujian Province, China). V. alginolyticus strain ATCC 17749^T^ was grown overnight in alkaline peptone water (APW) supplemented with 2% (wt/vol) NaCl at 37°C with vigorous shaking. The bacterial cells were pelleted by centrifugation and the supernatants discarded. The bacterial cells were washed three times with phosphate-buffered saline (PBS) before being resuspended to an approximate final concentration of 5 × 108 CFU/ml. The QIAamp DNA minikit (Qiagen) was used for DNA extraction. The quality and quantity of DNA were evaluated spectrophotometrically with the NanoDrop ND1000 (Thermo Scientific, Wilmington, DE). A concentration of 50 ng/µl was used for sequencing. V. alginolyticus ATCC 17749^T^ was sequenced at the Chinese National Human Genome Center (CNHGC) at Shanghai using a massively parallel pyrosequencing technology (Roche 454 GS FLX) with a shotgun. The data of this genome were annotated and validated using gene prediction tools, such as Glimmer 3.02, GeneMark, and the Z-curve program. tRNAscan (16) and RNAmmer and were used to identify tRNAs and rRNAs, respectively. One hundred twenty-two contigs (>500 bp) with a total size of 5,059,562 bp were assembled from 186,433 reads using Newbler software of the 454 suite package, providing 19.5-fold coverage. The average contig length was 45,174 bp, and the maximum contig length was 401,518 bp, with an overall G+C content of the V. alginolyticus genome assemblies being 44.6%.

The size of the complete genome of V. alginolyticus (ATCC 17749^T^) is 5,146,637 bp, with a coding sequence percentage of 86.1%. The G+C contents of the coding and intergenic regions are 45.6% and 39.0%, respectively. The total number of determined coding sequences (CDSs) is 4,722, with an average length of gene about 942 nucleotides. The genome of this strain revealed 105 tRNA and 34 rRNA genes.

In this work, the first complete genome of V. alginolyticus type strain ATCC 17749^T^ was elucidated. We hope this genome information provides positive contributions to the study of virulence factors and the development of new accurate diagnostic methods.

### Nucleotide sequence accession numbers.

The complete genome sequences of the chromosome have been deposited at DDBJ/EMBL/GenBank under the accession numbers CP006718 and CP006719.
